# On Gout: Its Cause, Nature, and Treatment

**Published:** 1841-10

**Authors:** 


### ART. XVII.

*On Gout: its Cause, Nature, and Treatment.* By JOHN PARKIN, Mem. of the Roy. Coll. of Surgeons, &c.—*London*, 1841. 8vo, pp. 140.

THE main object of this work is to "endeavour to ascertain if any theory can be proposed, which is not only sufficient to account for the origin of the disease (gout), and at the same time to explain the various anomalies that belong to all other theories, but which leads to the adoption of a mode of treatment as scientific and as successful as that adopted for the majority of diseases." By the term "cause," Mr. Parkin would be understood to refer to that primary or original cause, which produces the gouty diathesis, or morbid state of the system, and not those predisposing or exciting causes which merely favour or accelerate the development of a paroxysm, in those in whom the seeds of the disease have been already sown by the agency of another and more specific cause. The attempt to explain the "primary or original cause," the proximate cause of gout, or of any other disease, has at least the merit of courage. For our own parts we confess that, in our opinion, the labour that has from the earliest ages been bestowed upon the effort to discover the essence or intimate nature of gout, and perhaps of any other malady, would have been much more usefully exerted in the careful consideration of the rise, progress, and variation of the essential and apparent symptoms of disease, and the effect of remedies. We are not at all inclined to admit the validity of the very plausible and frequently repeated dogma, that he who knows nothing of the essence of disease can know but little, if anything, of the mode of curing it. If this were true, the practical utility of our art would be bounded by very narrow limits; for not to assume the knowledge we certainly do not at present possess, it must be admitted that the essence of disease, the *materies morbi*, still remains a mystery to us. In natural philosophy and medicine, it is often sufficient for all practical purposes that we should be acquainted with particular phenomena, although the causes which produce them may escape our closest scrutiny. "Sufficit si quid fiat intelligamus, etiamsi quomodo quidque fiat ignoremus," says Cicero. As we have no wish, however, to check the ambitious and aspiring efforts of those who are willing to cope with the difficulties of such an investigation, we proceed to enquire what light Mr. Parkin has thrown upon the "cause" of gout, confining the term within the limits that he assigns to it. He commences by giving us a critical sketch of the various speculations that have been indulged in by various writers upon the subject, all of which are dismissed as more or less obnoxious to doubt and objections—the learned authorities quoted by Mr. Parkin being chiefly those referred to in the medical dictionaries and cyclopædias. It is well known to every practitioner at all acquainted with the literature of his profession, that the doctrine of a morbid matter in the blood, as constituting the cause of gout, has had powerful supporters from the earliest ages, and that even in the present day this belief has many advocates.

"If, however," says Mr. Parkin, "we enquire into the nature of the morbid matter, we must draw a different conclusion to that of the ancient writers, who considered it to be either bile, phlegm, or blood, which latter they supposed was propelled into vessels which did not contain it at other times, a very natural inference for those who were ignorant that the vital fluid circulated in the arterial as well as the venous system. That bile, again, is not the cause of gout may be inferred from the fact, that when there is an obstruction to the natural flow of this secretion, and the arterial system becomes in consequence overloaded with it, no attack is witnessed. With respect to phlegm, we have in the first place no reason to believe that this excreted matter becomes absorbed into the system, or that, if present there, it would give rise to the effects in question, while we are certain that the presence of healthy blood in the capillaries will not account for the phenomena, inasmuch as they contain this fluid at all times. But, although the presence of the above matters in the blood is insufficient to account for the production of gout, it does not follow that there are no other substances capable of producing the disease, when present in the system.

"That the presence of an extraneous or morbid matter in the blood would be capable of giving rise to effects similar to those witnessed in this disease, we might presume, *à priori*, while the supposition is confirmed by what has been observed after the introduction of various poisonous substances into the animal frame." (pp. 13-4.)

Inasmuch as there are scarcely any limits to our presumption, we certainly " might presume" this fact " *a priori*;" but why we should so presume, we are as much at a loss to discover, as we are how, or in what manner, the presumption is at all strengthened by the examples brought forward by Mr. Parkin to show what nobody denies, that various morbid substances, when present in the system, give rise to irritation and inflammation, not only on the internal surface of the body, but also in the extremities. We require proof for the assertion, " that various poisonous elements, when present in the system, produce effects similar to those observed during attacks of this disease," gout. Mr. Parkin goes no further than to show that there is a slight resemblance between the symptoms produced by the presence of deleterious agents in the system, and the symptoms of the preliminary stage of gout; but he fails entirely to show such an identity of effect, as to justify the inference of identity of cause. Having "endeavoured to show," and as we think unsatisfactorily, that gout is produced by the operation of a poison in the system, Mr. Parkin proceeds to enquire, " what the nature of the poison is and whence it is derived ;" and from a train of, we think, equally loose and inconclusive speculation with that upon which he has tried to prove the presence of some poison in the system, he comes to the conclusion that gout is the product of malaria! A few brief comments upon the " nature" of gout are summed up by the following passage:

"We thus learn that gout is sometimes of an inflammatory, sometimes of a congestive, and sometimes of a nervous character; and that it assumes a variety of forms in different individuals and under different circumstances. It is, therefore, neither a purely inflammatory, nor yet a purely nervous disease, nor is it characterized by an affection of one particular part of the body, or one particular system of nerves; for it attacks every organ and tissue, and produces a disturbance in each part of the circulating system, and in every function of the body, the same in those which are under the direct control of the cerebro-spinal, as in those which are under the influence of the ganglionic nerves.

"To view gout, therefore, as Dr. Barlow and other writers have done, only 'as a constitutional disturbance of an inflammatory character, attended with local inflammation of a peculiar kind in one or more joints,' would exclude from our definition a series of effects evidently allied to the former, and produced by the same cause. The definition of Frank is not only more general, but also more correct; for he says, 'gout is a disease *sui generis*, which can assume the form of all diseases, and present itself under the character of a fever, an inflammation, an eruption, a flux, a retention, or a nervous affection.'" (pp. 87-8.)

In this statement we agree with our author.—Although we do not deny the force of many of the objections Mr. Parkin urges to the use of colchicum in the treatment of gout, we are by no means prepared with him to expunge it from our list of remedies. We sincerely hope that Mr. Parkin's opinion of the value of "carbonic acid gas" in the treatment of gout will bear the test of experience. He says that "it may be considered a specific in gout," and that it acts "by neutralizing or rendering inert the poison productive of the disease," and that "the first and most obvious result which has attended the administration of carbonic acid gas in gout is that of shortening the paroxysm to a much greater extent than that of any other remedy which I have seen administered, or of which I have heard mention. This has been an *invariable* result, as far as my own experience goes." Now without for a moment doubting the good faith with which Mr. Parkin makes this statement, we must observe that we are always a little sceptical when we hear of the "invariable" virtues of either medicines or of men. Mr. Parkin's views of the general treatment of gout are postponed, "as the want of time, and some important engagements, prevent him from entering fully into this part of his subject." We beg to submit, that as Mr. Parkin was not compelled to publish an unfinished book, his plea of "want of time," &c. does not justify his haste. We do not find anything in the volume so essentially necessary to the welfare of the public, or the instruction of the profession, as to have precluded Mr. Parkin from waiting until he could snatch a little more leisure from "important engagements," and give us a whole work instead of half a one. We are promised a "second part to the present work," containing the directions that Mr. Parkin considers proper for the "treatment of the different stages, as well as the different forms of gout." We can wait for it patiently, and we trust that the author will not again enter upon his undertaking, until he has sufficient leisure to fulfil the contract between himself and the purchasers of his book implied in his title-page.

---

